# Acinar Cell Cystadenoma: A Case Report and Literature Review

**DOI:** 10.7759/cureus.108791

**Published:** 2026-05-13

**Authors:** Karikal Chakaravarthi, Manojkumar Perumal, Kalaivani Palaniswamy, Mohamed Jaavidh Abdul Gani, Naganath Babu Obla Lakshmanamoorthy

**Affiliations:** 1 Department of Surgical Gastroenterology, SRM Medical College Hospital and Research Centre, SRM Medical College SRM Institute of Science and Technology, Chengalpattu, IND; 2 Department of General Surgery, SRM Medical College Hospital and Research Centre, SRM Medical College SRM Institute of Science and Technology, Chengalpattu, IND; 3 Department of Pathology, SRM Medical College Hospital and Research Centre, SRM Medical College SRM Institute of Science and Technology, Chengalpattu, IND

**Keywords:** acinar cell cystadenoma, acinar cystic transformation of pancreas, cystic lesion of the pancreas, digestive organs/liver/pancreas, endoscopic ultrasound-guided biopsy, intraductal papillary mucinous neoplasm, modified heidelberg technique, pancreas, rare tumors

## Abstract

Acinar cell cystadenoma (ACA) of the pancreas, also known as acinar cystic transformation, is a rare benign cystic lesion with no established malignant potential. Preoperative distinction from mucinous and other premalignant cystic neoplasms remains unreliable. A 39-year-old Indian woman presented with persistent upper abdominal pain. Cross-sectional imaging demonstrated a 4 × 4 cm multiloculated cystic lesion in the pancreatic head with septations and peripheral calcifications and without ductal communication, findings suggestive of a mucinous cystic neoplasm. Serum tumor markers were normal. Because of the symptomatic presentation and radiological concern for premalignant pathology, pancreaticoduodenectomy was performed. Histopathological examination confirmed ACA, with no evidence of dysplasia or malignancy. The postoperative course was uneventful, and the patient remained asymptomatic on follow-up with no evidence of recurrence. This case highlights the diagnostic challenges associated with ACA and the risk of overtreatment due to its radiological similarity to premalignant cystic neoplasms.

## Introduction

Acinar cell cystadenoma (ACA), also known as acinar cystic transformation, is a rare pancreatic cystic lesion with fewer than 200 cases reported in the literature to date, which was first described as an incidental autopsy finding in 2002 [1]. Almost half of these were incidentally identified, and the rest were presented with abdominal pain. Most reported cases occur in females aged 18-57 years. ACA is commonly associated with the pancreatic head [2]. ACAs are multilocular or unilocular, and their size ranges from 1.8 to 15 cm [3]. Preoperative diagnosis is often challenging. It is commonly diagnosed after surgical resection. Histologically, multilocular ACAs are lined by patches of acinar and ductal epithelium [4]. We report a case of a 39-year-old Indian woman with abdominal pain and a cystic neoplasm involving the pancreatic head who underwent a classical Whipple procedure. Postoperative histopathological examination revealed an ACA. This case highlights the diagnostic challenges associated with ACA and the risk of overtreatment due to its radiological similarity to premalignant cystic neoplasms.

## Case presentation

**Figure 1:**
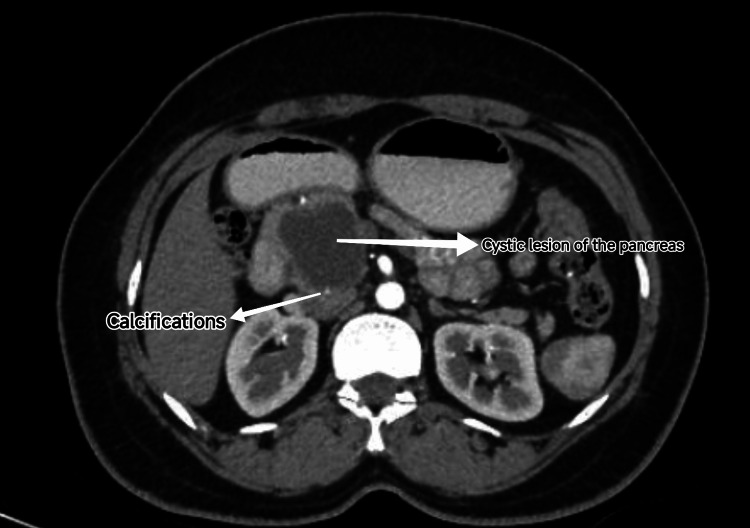
Contrast-enhanced CT abdomen Contrast-enhanced CT abdomen shows a thin-walled, multiloculated cystic lesion measuring 4 x 4 cm over the head and uncinate process of the pancreas, with peripheral septations and tiny calcifications. CT: computed tomography

A 39-year-old Indian woman without any known comorbidities presented to the surgical gastroenterology outpatient clinic at SRM Medical College Hospital and Research Centre, Chengalpattu, in April 2025 with a history of intermittent upper abdominal pain for six months, which was insidious in onset. There was no history suggestive of obstructive jaundice. There was no significant past medical or surgical history. No significant family history was noted. Clinical examination of the abdomen was normal. An abdominal ultrasonogram revealed a cystic lesion measuring 4 × 4 cm in the head and uncinate process of the pancreas. Cross-sectional CT imaging confirmed a thin-walled, multiloculated cyst with peripheral septations and calcifications, without communication with the main pancreatic duct, raising suspicion for a mucinous cystic neoplasm (Figure 1). Magnetic resonance cholangiopancreatography revealed well-defined multiloculated cystic lesions measuring 4 × 4 cm in the head and uncinate process of the pancreas, with a few thin internal septations and without communication between the main pancreatic duct and the lesion.

**Figure 2:**
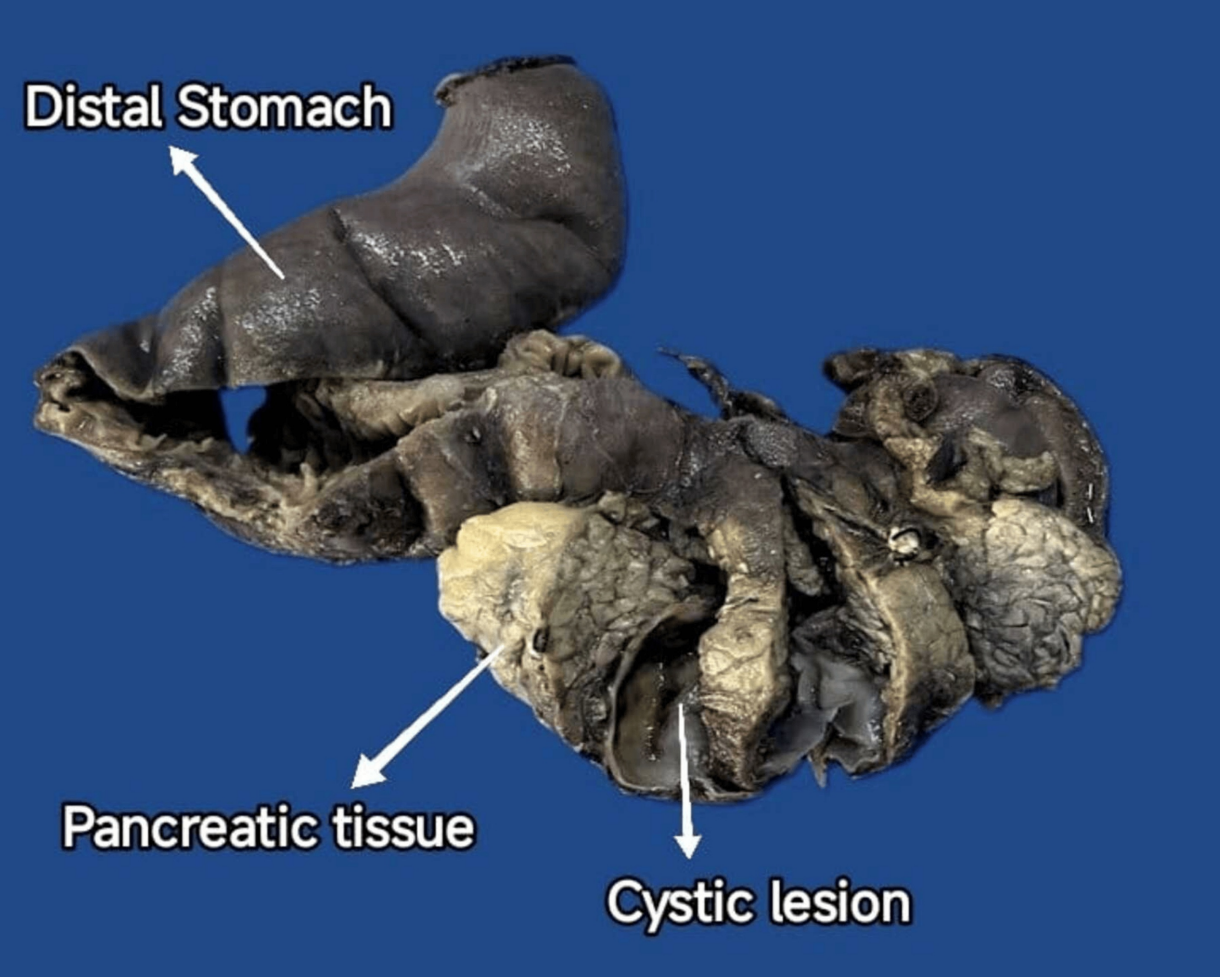
Gross specimen showing a multiloculated cystic lesion in the pancreatic head without solid components

Oesophagogastroduodenoscopy was normal. All routine blood investigations, including serum amylase and lipase, were normal. Tumor markers, including carcinoembryonic antigen (<5.0 ng/mL) and carbohydrate antigen 19-9 (<37 U/mL), were within normal ranges. Because of the pain symptoms and imaging evidence suggestive of mucinous cystic neoplasm, it was decided to perform surgery; endoscopic ultrasound-guided fine needle aspiration was deferred. The patient underwent a classical Whipple procedure. Pancreaticojejunostomy was performed using a modified Heidelberg technique with placement of a pancreatic stent. Intraoperatively, a 4 x 4 cm cystic mass was noted in the head and uncinate process. Cystic fluid analysis was negative for malignant cells and mucin. Grossly, the cut surface of the lesion revealed multiple papillary excrescences (Figure 2).

**Figure 3:**
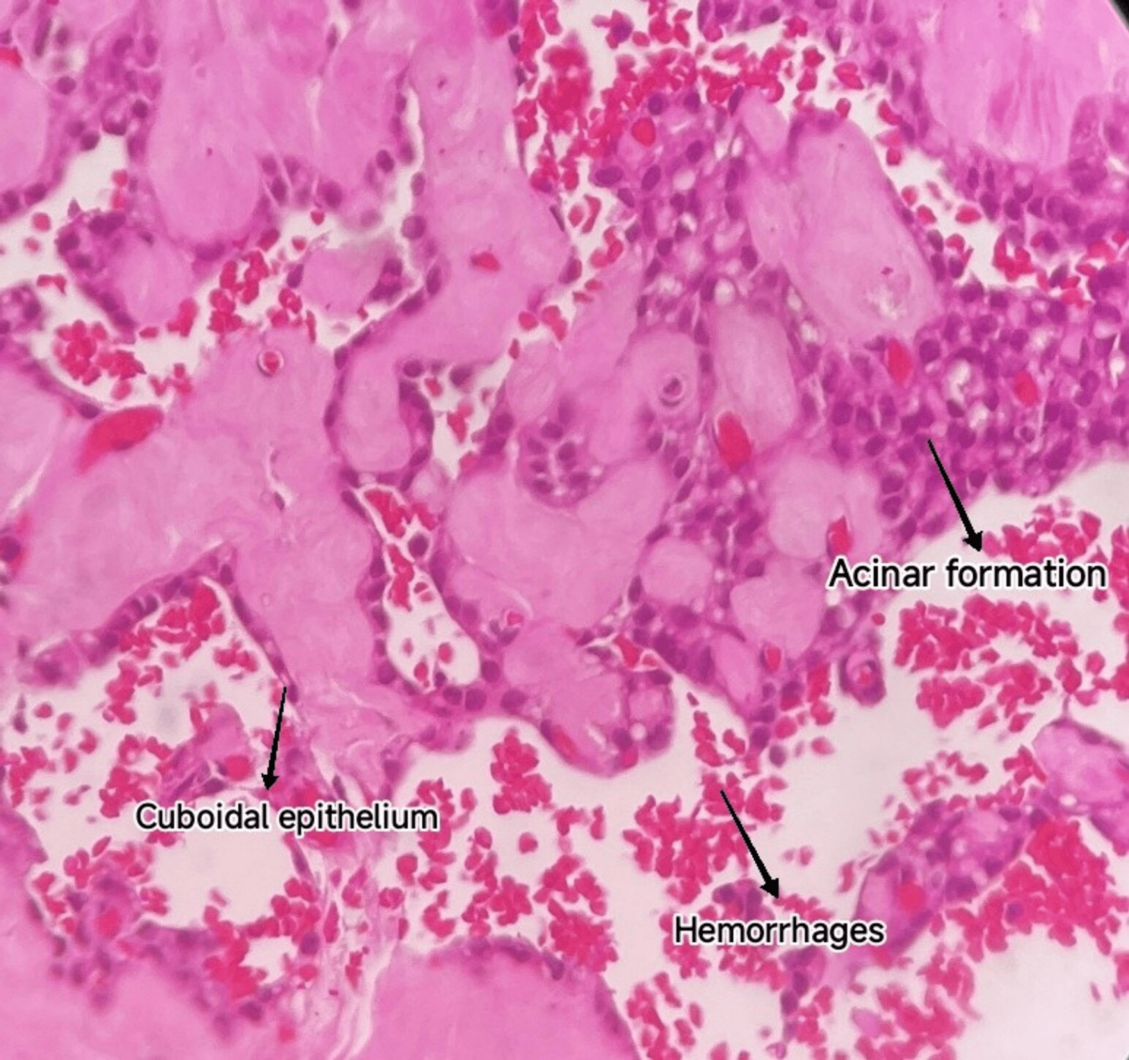
Histopathology showing cyst wall lined by cuboidal epithelium with acinar differentiation (hematoxylin and eosin stain, ×40 magnification)

Histopathological examination revealed a cystic lesion composed of a fibrotic wall lined by stratified cuboidal epithelium. The lining epithelium demonstrated features of pancreatic acinar differentiation. Focal areas showed pseudopapillary architecture, with epithelial cells arranged in one to two layers. The cyst lumen contained areas of hemorrhage. Occasional cells exhibited intracytoplasmic vacuolation. No evidence of cytological atypia, mitotic activity, or necrosis was identified. No associated low-grade pancreatic intraepithelial neoplasia or high-grade dysplasia was observed in the adjacent pancreatic parenchyma. All surgically resected margins were free of lesions. The examined pancreaticoduodenal lymph node showed reactive changes without evidence of metastasis, confirming the diagnosis of ACA (Figure 3).

The postoperative course was uneventful, with no evidence of pancreatic fistula or hemorrhage. The patient is on regular follow-up once every six months. No recurrence was noted to date.

## Discussion

ACA is an exceedingly rare, benign cystic lesion of the pancreas, originally described by Albores-Saavedra in 2002 [1]. The 2019 WHO Digestive System Tumor classification redefined this entity as a non-neoplastic cystic lesion, distinct from acinar cell carcinoma and cystadenocarcinoma [2]. Originally recognized as a non-neoplastic cyst and referred to as "acinar cystic transformation," ACA was subsequently assigned the “-oma” nomenclature, despite the absence of strong evidence indicating neoplastic behavior [3]. Most reported cases occur in females aged 18-57 years (mean 43 years); a few cases were also reported in the pediatric age group. While many are incidentally discovered, some may develop symptoms like abdominal pain [3,5,6]. Radiologically, these lesions typically appear as multiloculated cysts within the pancreatic head, averaging 4.8 cm in size [7]. Imaging modalities, including CT and MRI, lack specificity for definitive identification.

The principal differential diagnoses include mucinous cystic neoplasms, serous cystic neoplasms, and branch-duct intraductal papillary mucinous neoplasms [8]. Although endoscopic ultrasound-guided cyst fluid analysis and molecular profiling can aid in preoperative characterization, their role is limited in symptomatic cases requiring surgery, where management is unlikely to change [5]. Delavaud et al. proposed imaging features for distinguishing ACA, such as (a) five or more cysts, (b) clustered peripheral small cysts, (c) presence of cyst calcifications, and (d) absence of communication with the main pancreatic duct [9]. The European study group suggested guidelines for the surgical resection of cystic tumors of the pancreas; i.e., surgical excision remains appropriate when symptoms or suspicious imaging features are present, as in the current case [10]. The histological hallmark is cyst lining with acinar and ductal epithelial elements, typically immunoreactive for trypsin, chymotrypsin, and cytokeratin 7. Endoscopic ultrasound-guided through-the-needle biopsy, or transgastric endoscopic puncture of a pancreatic cyst, may provide a definitive preoperative diagnosis prior to radical surgery. The diagnostic yield of a preoperative biopsy, however, is highly dependent on the sampling of the representative tissue. When the sample is limited, the characteristic acinar epithelial lining may be missed, potentially leading to misclassification as chronic pancreatitis rather than recognition of ACA. Once the diagnosis of ACA is made, it can be managed conservatively with regular clinical and imaging follow-up at regular intervals and imaging (CT/MRI) at defined intervals (e.g., 6-12 months). Surgery is indicated if the patient becomes symptomatic [5,11-13]. No standardized surveillance exists for ACA, given its benign nature.

## Conclusions

ACA is a rare benign pancreatic lesion that can closely resemble other premalignant and malignant cystic neoplasms on imaging. In the absence of reliable preoperative diagnostic tools, surgical resection is often undertaken. This case highlights the diagnostic challenge posed by ACA presenting as a mucinous cystic neoplasm in a symptomatic patient, leading to pancreaticoduodenectomy. It underscores the need for improved preoperative diagnostic strategies to avoid unnecessary radical surgery.

## References

[1] Albores-Saavedra J (2002). Acinar cystadenoma of the pancreas: a previously undescribed tumor. Ann Diagn Pathol.

[2] Nagtegaal ID, Odze RD, Klimstra D (2020). The 2019 WHO classification of tumours of the digestive system. Histopathology.

[3] Singhi AD, Norwood S, Liu TC (2013). Acinar cell cystadenoma of the pancreas: a benign neoplasm or non-neoplastic ballooning of acinar and ductal epithelium?. Am J Surg Pathol.

[4] Rift CV, Hasselby JP, Hansen CP, Federspiel B (2020). Acinar cystic transformation of the pancreas: report of a case and a review of the literature. Pathol Res Pract.

[5] Conti Bellocchi MC, Manfrin E, Brillo A (2023). Rare pancreatic/peripancreatic cystic lesions can be accurately characterized by EUS with through-the-needle biopsy-a unique pictorial essay with clinical and histopathological correlations. Diagnostics (Basel).

[6] Mitra S, Kalra M, Purkait S (2022). Congenital acinar cystic transformation of the pancreas with proximal jejunal atresia and hepatic iron overload: an autopsy case. Fetal Pediatr Pathol.

[7] Mattiolo P, Wang H, Basturk O (2023). Comprehensive characterisation of acinar cystic transformation of the pancreas: a systematic review. J Clin Pathol.

[8] Sergi W, D'Ugo S, Libia A (2023). Symptomatic acinar cell cystadenoma of the pancreas. J Surg Case Rep.

[9] Delavaud C, d'Assignies G, Cros J (2014). CT and MR imaging of multilocular acinar cell cystadenoma: comparison with branch duct intraductal papillary mucinous neoplasia (IPMNs). Eur Radiol.

[10] (2018). European evidence-based guidelines on pancreatic cystic neoplasms. Gut.

[11] Zhong XY, Liang ZJ, Lan ML, Xu XG, Yuan L, Zeng JX (2025). Acinar cystic transformation of the pancreas: a rare case report. World J Clin Cases.

[12] Orr J, Lockwood R, Roberts J, Shi C, Yachimski P (2018). EUS and confocal endomicroscopic diagnosis of pancreatic acinar cell cystadenoma. Gastrointest Endosc.

[13] Steinkraus KC, Mühlberger M, Schmidt SA, Kornmann M (2023). Acinar cystic transformation of the pancreas-a rare case in a young patient. J Surg Case Rep.

